# Recommendations for the implementation and conduct of multidisciplinary team meetings for those providing endometriosis and adenomyosis care - a Delphi consensus of the European Endometriosis League (EEL)

**DOI:** 10.52054/FVVO.16.3.038

**Published:** 2024-09-30

**Authors:** L Burla, DR Kalaitzopoulos, N Samartzis, S Khazali, A Bokor, SP Renner, G Hudelist, AS Constantin, SD Schäfer, J Nassif, A Naem, J Keckstein, H Krentel, EEL MDT meeting Consensus Group, C Becker, V Bindra, N Bourdel, E Brătilă, S Burghaus, M Ćorić, A Daniilidis, B Diaz de la Noval, M Eberhard, K Galczynski, A Gisselmann Egekvist, S Imboden, S Johnson, S Kamm, B Kraemer, AC Lou-Mercadé, N Magunska, K Manolopoulos, D Miligkos, G Mitroi, M Mormont, M Mueller, A Nap, M Ormos, Y Osuga, C Polli, VP Ramos Barrientos, N Rohloff, K-W Schweppe, LM Senturk, M Sillem, J Stöckl, D Tschida, D Tsepov, S Verta, J Vitols, M Wölfler

**Affiliations:** Nuffield Department of Women’s and Reproductive Health, University of Oxford, Oxford, UK; Advanced Centre for Endometriosis (ACE), Apollo Hospitals, Hyderabad, India; CHU Clermont-Ferrand, Clermont-Ferrand, France; University of Medicine and Pharmacy Carol Davila, Bucharest, Romania; Department of Gynecology and Obstetrics, Erlangen University Hospital, University Endometriosis Center for Franconia, Friedrich-Alexander University Erlangen-Nürnberg, Germany; Department of Gynecologic Surgery, University Clinic of Zagreb, Croatia; Papageorgiou General Hospital, Aristotle University of Thessaloniki, Greece; Gynaecology and Obstetrics, Hospital Universitario Central de Asturias, Oviedo, Spain; Department of Gynecology and Obstetrics, Cantonal Hospital of Winterthur, Winterthur, Switzerland; Faculty of Medical and Health Sciences, University of Siedlce, Poland; Department of Obstetrics and Gynecology, Aarhus University Hospital, Aarhus, Denmark; Department of Gynecology & Gynecological Oncology, Inselspital, University Hospital Bern, Bern, Switzerland; Princess Anne Hospital, Southampton, UK; Clinic for Gynecology and Obstetrics, Limmattal Hospital, Switzerland; University Hospital for Women, Tübingen, Germany; Obstetrics and Gynecology, Hospital Universitario Lozano Blesa, Zaragoza, Spain; Department of Gynecology and Gynecological endoscopy, Hospital Dr. Shterev, Sofia, Bulgaria; Center for Fertility and Endometriosis, Offenbach, Germany; Southampton University Hospitals, Southampton, UK; Life Memorial Hospital, Bucharest, Romania; Clinique de Valère, Sion, Switzerland; Department of Gynecology & Gynecological Oncology, Inselspital, University Hospital Bern, Bern, Switzerland; Department of Gynecology and Obstetrics, Radboudumc, Nijmegen, the Netherlands; Hospital Baden, Baden, Switzerland; Obstetrics and Gynecology, the University of Tokyo, Japan; EOC - Ente ospedaliero cantonale, Ospedale Civico Lugano, Switzerland; Clínica Montesur, Santiago de Surco, Peru; Endo Health GmbH, Germany; Stiftung Endometriose-Forschung, Westerstede, Germany; Istanbul University-Cerrahpasa, Istanbul, Turkey; Department of Gynecology and Obstetrics, Saarland University Hospital, Homburg, Germany; Department of Gynecology and Obstetrics, Osnabrück Hospital, Osnabrück, Germany; LKH Hochsteiermark, Leoben, Austria; LEAPS (London Gynaecology and Advanced Pelvic Surgery Centre), HCA Healthcare UK, London, UK; Department for Gynecology and Obstetrics, Hospital Lucerne, Lucerne, Switzerland; JV Clinic, Riga, Latvia; Department of Obstetrics and Gynecology, Medical University of Graz, Austria; Department of Gynecology and Obstetrics, Hospital Schaffhausen, Schaffhausen, Switzerland; Department of Gynecology, University Hospital of Zurich, Zurich, Switzerland; Center for Endometriosis and Minimally Invasive Gynecology (CEMIG London), HCA The Lister Hospital, London, United Kingdom; Department of Obstetrics and Gynecology, Faculty of Medicine, Semmelweis University, Budapest, Hungary; Department of Gynecology and Obstetrics, Hospital Böblingen, Klinikverbund-Suedwest, Klinikum Sindelfingen-Böblingen, Böblingen, Germany; Department of Gynecology, Center for Endometriosis, Hospital St. John of God, Vienna, Austria; Department of Gynecology and Obstetrics, Saarland University Hospital, Homburg, Germany; Department of Gynecology and Obstetrics, Clemenshospital Muenster, Muenster, Germany; Division of Minimally Invasive Surgery, Department of Obstetrics and Gynecology, Baylor College of Medicine & Texas Children’s Hospital, Houston, Texas, USA; Department of Obstetrics, Gynecology, Gynecologic Oncology and Senology, Bethesda Hospital Duisburg, Duisburg Germany; Faculty of Mathematics and Computer Science, University of Bremen, Bremen, Germany; Endometriosis Clinic Dres. Keckstein, Villach, Austria; University Ulm; Ulm, Germany

**Keywords:** Endometriosis, adenomyosis, endometriosis multidisciplinary team meetings, multidisciplinary team meetings, multidisciplinary teams, multidisciplinary endometriosis board

## Abstract

**Background:**

The treatment of endometriosis and adenomyosis requires a complex, multidisciplinary approach. Some centres have established multidisciplinary teams (MDT) and regular meetings. There are currently no international data or recommendations.

**Objectives:**

To examine existing MDT meetings and define consensus recommendations to support implementation and conduct.

**Materials and Methods:**

Online questionnaires were sent through the European Endometriosis League (EEL) based on a Delphi protocol. After a literature review and assessment of existing MDT meetings, essential aspects for consensus statements were identified. The consensus statements were evaluated using a 5-point Likert scale with the possibility to modify them. Results were analysed between rounds and reported to the respondents. Consensus, defined as ≥70% agreement, concluded the Delphi process when achieved in the majority of statements.

**Main outcome measures:**

Prevalence and type of existing MDT meetings and recommendations.

**Results:**

In round 1, 69 respondents participated, with 49.3% (34) having an MDT meeting at their institutions, of which 97% are multidisciplinary. 50 % meet once a month and 64.7% indicated that less than 25% of their patients are discussed. Throughout the three rounds, 47 respondents from 21 countries participated. During the process, 82 statements were defined, with an agreement of 92.7% on the statements.

**Conclusions:**

This study assessed existing MDT meetings for endometriosis and adenomyosis and developed recommendations for their implementation and conduct. The consensus group supports the strengths of MDT meetings, highlighting their role in offering guideline-based, multidisciplinary, and personalised care.

**What is new?:**

This study presents the first international data and recommendations on MDT meetings for endometriosis and adenomyosis.

## Introduction

Endometriosis is a hormone-dependent inflammatory disease and is defined as extra- uterine endometrium-like tissue. This condition affects approximately 10% of reproductive-age women. Endometriosis is typically associated with dysmenorrhoea, dyspareunia, dyschezia, cycle-independent pelvic pain and a variety of other possible symptoms and can cause infertility (Zondervan et al., 2020). Adenomyosis, endometrium-like tissue within the myometrium, often co-occurs with endometriosis although the exact relationship is not yet fully understood (Guo, 2020). These conditions can have a profound impact on affected women’s wellbeing, leading to a substantial socioeconomic and healthcare burden (Della Corte et al., 2020; Simoens et al., 2012).

The overlap of clinical manifestations with other gynaecological and non-gynaecological diseases and the lack of awareness among healthcare providers and the wider community can lead to a delayed diagnosis and inappropriate therapy (Agarwal et al., 2019).

Diagnosis and treatment of endometriosis and adenomyosis are complex and require a high level of specialisation from all involved physicians from various disciplines, such as general gynaecology, gynaecological surgery, reproductive medicine, radiology, and in some cases, urologists and visceral and thoracic surgeons (Chapron et al., 2020; Becker et al., 2022; Krentel et al., 2022). Peri-therapeutic management and follow-up quality indicators, such as hospitalisation duration, patient satisfaction, and complications, have only been sporadically examined (Weyl et al., 2023; Hudelist et al., 2022; Turco et al., 2020). In a few countries, validation and governance programs for endometriosis centres, including the recommendation for MDTs, have been implemented. Nevertheless, the otherwise widespread absence of national or international quality assurance is noticeable.

Based on experience, relevant differences among healthcare providers in terms of treatment-related parameters still exist even though efforts to minimise such differences are increasing (Krentel et al., 2022).

MDTs have long played a crucial role in treatment decisions for other chronic and oncological diseases (Pillay et al., 2016; Basta et al., 2016). In gynaecology, MDTs are already used in different areas, such as urogynaecology, and have proven to be effective (Gopinath and Jha, 2015). Similarly, some centres have also introduced regular MDT meetings for endometriosis, which are referred to as endometriosis MDT meetings or multidisciplinary endometriosis boards (MEB) as described by several groups of researchers (Ugwumadu et al., 2017; Bolze et al., 2019; Allaire et al., 2020).

The developments of recent years have proposed a fundamental change in endometriosis management by emphasising the pre-therapeutic assessment of the complexity of the disease using transvaginal ultrasound (TVUS) and magnetic resonance imaging (MRI). This type of assessment promotes collaboration among different medical specialties and allows for a tailored multidisciplinary approach, including pre- and post-therapeutic classification of the disease (Becker et al., 2022; Keckstein et al., 2023; Maciel et al., 2023).

At present, data describing the current status of existing endometriosis MDT meetings are scarce. Additionally, guidelines for the conduct of endometriosis MDT meetings are currently lacking. The present consensus addresses this gap by providing an analysis of all aspects regarding endometriosis MDT meetings.

## Materials and methods

### Recruitment of respondents

Respondents were invited to participate through the members network of the European Endometriosis League (EEL). The invitations included a detailed overview of the study protocol, which outlined the Delphi consensus process, and indicated that the project was of a scientific nature. EEL members were chosen as the respondent recruitment pool as its members focus on endometriosis, encompass both academic and clinical interests, and represent various countries with diverse healthcare systems ranging from private practices to academic institutions. The aim was to assemble a diverse group with a shared focus to generate international and generalisable recommendations independent of existing certification bodies.

### Delphi Survey

The Delphi survey was conducted using an online tool (SurveyMonkey, Palo Alto, California, USA). This approach ensured the anonymity of the respondents among each other during the voting and commenting stages. After agreeing to participate, respondents were granted access to each Delphi round via the email addresses they provided. In total, three rounds starting in March 2023 were conducted online without a meeting of the respondents. The respondents had the email contact of the author team who were available for any questions. The recruitment phase and first round lasted for three months. Following that round, each additional round lasted for four weeks with a four-week break in between. During each round, two reminders were sent. A feedback email with the results was sent to every respondent individually after each round. Each respondent received an overview of the total results and her/ his individual responses for comparison.

Demographic data and respondent’s experience were collected, but this information was not shared with the other respondents (Table I).

**Table I:** Demographics of the 47 respondents who completed the Delphi procedure. Any discrepancies in the number of responses are indicated in the left column.

	Overall, n = 47
Mean age in years (SD)	48 (10)
Gender	
Female	38.3% (n=18)
Male	61.7% (29)
Type of institution	
University Hospital	44.7 % (21)
Referral Centre but not academic	27.7% (13)
Regional Hospital	2.1% (1)
Private Clinic or Practice	25.5% (12)
Position	
Head of Department / Clinic	27.7% (13)
Deputy Head of Department / Clinic	6.4% (3)
Senior Consultant / Attending Physician	42.6% (20)
Consultant / Attending Physician	8.5% (4)
Independent Specialist (e.g. Working in Private Practice)	14.9% (7)
Working in a certified endometriosis centre	
Yes	55.3% (26)
Years of treating endometriosis patients	
<5	2.1% (1)
5 - 10	34% (16)
11 - 20	36.2% (17)
21 - 30	19.2% (9)
>30	8.5% (4)
Specialty regarding endometriosis treatment	
General Gynaecology	40.4% (19)
Gynaecologic Surgery	85.1% (40)
Diagnostics, Ultrasonography	46.8% (22)
Reproductive Medicine	19.2% (9)
Institutional general caseload per year	
<100	4.3% (2)
100 - 250	17% (8)
251 - 500	40.4% (19)
501 - 1000	25.5% (12)
>1000	12.8% (6)
Institutional surgical caseload per year (46)	
<50	10.6% (5)
50 - 100	21.3% (10)
101 - 250	42.6% (20)
251 - 500	17% (8)
>500	8.5% (4)

### Preparation and preselection of aspects and Delphi round 1

We based the design and conduct of the present Delphi procedure on various methodological papers in addition to previously published Delphi consensus (Jünger et al., 2017; Beiderbeck et al., 2021; Boulkedid et al., 2011; Martin et al., 2020; Müller et al., 2021; Nasa et al., 2022). To initiate the Delphi process, the authors conducted a comprehensive literature search using the search tool PubMed® in February 2023. On one hand, searches were conducted for existing recommendations and/or guidelines related to the establishment and operation of MDTs and MDT meetings in general, such as those for different chronic conditions or tumour MDT meetings. Search terms included ((Chronic Disease) OR (Cancer) OR (Tumour)) AND ((Recommendations) OR (Guidelines)) AND ((Multidisciplinary board) OR (Multidisciplinary team meeting) OR (MDT)).

In the next step, data concerning endometriosis MDTs were searched. Search terms included ((Endometriosis) OR (Adenomyosis)) AND ((Board) OR (multidisciplinary board) OR (multidisciplinary endometriosis board) OR (MEB) OR (multidisciplinary team) OR (Multidisciplinary team meeting) OR (MDT)). The latter literature search revealed that specific literature addressing endometriosis MDTs was exceedingly sparse, which emphasised the need for a Delphi process approach. In Delphi round 1, the aim was to identify relevant aspects for endometriosis MDT meetings. Based on the search results and current guidelines, the authors defined the concept and derived aspects that could be relevant in the implementation and conduct of endometriosis MDT meetings (Becker et al., 2022; Kalaitzopoulos et al., 2021). A pretest was conducted within the group of authors after drafting Delphi round 1. The aspects were divided into several topics: (1) General Aspects, (2) MDT Structure, (3) Institutions, (4) Patient Selection, (5) Imaging Modalities, and (6) Classification.

These aspects were sent to the respondents of the consensus group in Delphi round 1. They could assess these criteria using a 5-point Likert scale: (1) not relevant, (2) barely relevant, (3) moderately relevant, (4) relevant, and (5) very relevant. If a specific aspect was considered relevant or very relevant by ≥ 50% of respondents, it was carried over to Delphi round 2 for further examination. The respondents had the opportunity to modify the aspects proposed by the authors or introduce their own aspects.

Additionally, it should be noted that in some centres, endometriosis MDTs already exist. The authors collectively decided that the consensus group should include both doctors with and without experience with endometriosis MDTs. This was considered important to create consensus statements that were as broadly applicable as possible while also providing new and independent inputs. Thus, in Delphi round 1, existing MDT meetings were examined in a survey, and these findings along with the aspects assembled by the authors were incorporated into the further Delphi process for the consensus statements (Supplement 1).

### Delphi rounds 2 and 3

In round 2, all respondents were presented with multiple choice questions, which had been compiled by the authors. These questions aimed to investigate the aspects gathered in round 1 in more detail. Each question included a comment feature for feedback and suggestions, and an additional comment field at the end of the survey was present. The multiple choice answers that were selected by ≥ 50% of the respondents at the end of round 2 were used by the authors to define the consensus statements (Supplement 2).

In round 3, respondents could rate the consensus statements on a 5-point Likert scale (strongly disagree, disagree, neither agree nor disagree, agree, strongly agree). Each statement had a comment feature for correction proposals. The Delphi consensus was concluded when the majority of consensus statements were approved by agreement or strong agreement ≥ 70% (Supplement 3).

### Considerations regarding the term ‘Endometriosis multidisciplinary team meeting’

In this work, no distinction was made regarding different endometriosis MDT meetings (size, country, criteria of a certification society, and other factors). We refer to regularly scheduled team meetings, either conducted by a single centre or multiple centres (multi-clinic), onsite and/or online.

The abbreviation “MDT” generally stands for “multidisciplinary team”; however, in this work, from this point onward, it will be used for simplicity to refer to an “endometriosis multidisciplinary team meeting”. Any exceptions to this will be explicitly highlighted. Additionally, the term “endometriosis MDT” is intended to encompass cases of both endometriosis and adenomyosis.

As the intention is to provide general recommendations that are practical for healthcare professionals regardless of whether they work in private practice or an academic centre, a distinction was made in the consensus statements: ‘To have/ be part of an MDT’ implies that the centre, when appropriate, should either have its own MDT or participate on a regularly scheduled basis in an MDT, such as in the case of multi-clinic MDTs. ‘To have access to an MDT’ means that the centre has contact with an MDT, and participation can occur when necessary.

### Statistical analyses

Statistical analyses were performed with IBM SPSS Statistics 27 (Endicott, New York, USA). Summary statistics for categorical variables were presented as numbers and percentages, while continuous variables were expressed as means along with their corresponding standard deviations (SD).

## Results

### Panel of respondents

**Figure 1:**
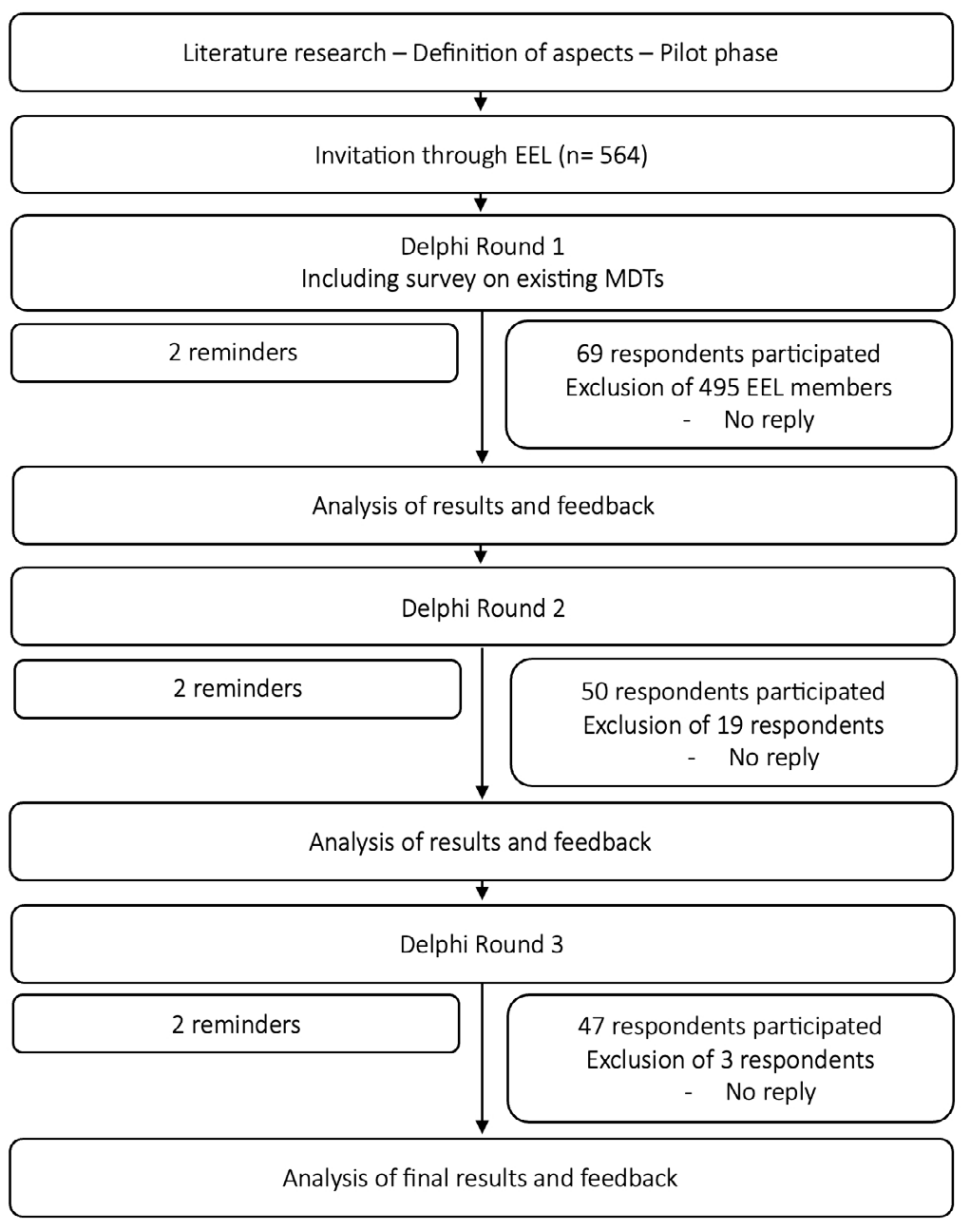
Flowchart showing the recruitment and engagement process of respondents.

The invitation to participate was sent to 564 EEL members; 495 EEL members did not respond to the invitation and were excluded. In round 1, 69 respondents participated. Across all rounds up to and including round 3, 47 respondents participated. The flowchart for participation across the rounds in addition to exclusions and their reasons can be found in Figure 1. The 47 respondents who participated up to and including round 3 were from 21 countries (Austria, Bulgaria, Croatia, Denmark, France, Germany, Greece, Hungary, India, Italy, Japan, Latvia, The Netherlands, Peru, Poland, Romania, Spain, Switzerland, Turkey, United Kingdom and the United States of America). 38.3% of these respondents were female, and almost 45% were working in university hospitals, while the other respondents were working in non-academic tertiary referral hospitals, private hospitals and private ambulatory practices. All respondents were specialists in gynaecology and endometriosis treatment with years of experience. Further details can be found in Table I.

### Current status of existing MDTs

Thirty-four (49.3%) of the 69 respondents from round 1 reported to already participate in a MDT at their workplace and thus took part in the survey on existing MDTs in round 1. Further details regarding the assessment of existing MDTs can be found in Table II. Table III provides an overview of the use of various classifications/scores within the existing MDTs, categorised by country.

**Table II:** Survey results from Delphi round 1 regarding current status of existing MDTs. Thirty-four (49.3%) of respondents declared that they have an MDT at their work place.

(n=respondents answered question)	% (n)
Cases discussed at the MDT compared to total volume (34)	
<25%	64.7% (n=22)
≈25-50%	32.4% (11)
≈ 50-75%	2.9% (1)
Surgical or conservative cases discussed (33)	
Mainly surgical	78.8% (26)
Balanced	21.2% (7)
Cases discussed pre- and post-therapy (33)	75.8% (25)
MDT frequency (34)	
>1/week	2.9% (1)
1/ week	14.7% (5)
1/ 2 weeks	17.6% (6)
1/ month	50.0% (17)
<1/month	14.7% (5)
Opportunity to register external cases (34)	52.9% (18)
Presenter of cases (33)	
Registering doctor	81.8% (27)
All cases presented by the same doctor in charge for the board	18.2% (6)
Multidisciplinary team meeting (different medical specialties) (33)	97.0% (32)
Multiprofessional MDT (other health care professions) (33)	63.6% (21)
Health care professionals usually present (33)	
Gynaecologic Surgeon	100.0% (33)
Radiologist	81.8% (27)
General/Visceral Surgeon	72.7% (24)
Reproductive Specialist	63.6% (21)
Urologist	51.5% (17)
Pain Specialist	39.4% (13)
Endometriosis Nurse	33.3% (11)
Physiotherapist	27.3% (9)
Nutritionist	18.2% (6)
Pathologist	15.2% (5)
Other	33.3% (11)
Ultrasound imaging shown at the MDT (33)	78.8% (26)
MR imaging shown at the MDT (33)	87.9% (29)
Intraoperative imaging shown at the MDT (33)	
Yes, in special cases	69.7% (23)
MR imaging shown by radiologist (33)	72.7% (24)
Sonographer also the surgeon (33)	
Yes, in most cases	72.7% (24)
Classifications used at the MDT (33)	
#Enzian	84.8% (28)
rASRM	75.8% (25)
EFI	42.4% (14)
The AAGL 2021 Endometriosis Classification	12.1% (4)
Other	18.2% (6)
Classification given pre- and post-therapy (33)	63.6% (21)
Definition of a follow-up at the MDT (33)	75.8% (25)
Any form of data collection at the MDT (32)	65.6% (21)
MDT helpful for teaching (33)	100.0% (33)

**Table III:** An overview of the use of different classifications/scores within the existing MDTs, categorised by countries. On the left, the number of respondents who provided input on the question and their existing MDT is listed next to the country. On the right, the classification is presented, along with the percentage of times it was mentioned across the countries, in cases where multiple respondents were from the same country. Each MDT can naturally use multiple classifications (dPEI = Deep Pelvic Endometriosis Index, EFI = Endometriosis Fertility Index, VNESS = Visual Numeric Endometriosis Surgical Staging).

	Overall respondents, n = 33
Austria (n respondents=3)	#Enzian (100%) rASRM (33.3%)
Bulgaria (1)	#Enzian rASRM
Croatia (2)	EFI (50%) #Enzian (50%) rASRM (100%)
Denmark (1)	EFI #Enzian
France (2)	AAGL 2021 (100%) dPEI (50%) EFI (100%) #Enzian (100%) rASRM (100%)
Germany (9)	EFI (33.3%) #Enzian (100%) rASRM (77.8%)
Hungary (1)	EFI #Enzian rASRM
Netherlands (2)	EFI (50%) #Enzian (50%) rASRM (50%)
Romania (1)	EFI #Enzian rASRM
Spain (1)	No specific Classification: description of Localisation
Switzerland (6)	EFI (66.7%) #Enzian (100%) rASRM (83.3%)
United Kingdom (4)	AAGL 2021 (50%) #Enzian (50%) rASRM (100%) VNESS (25%)

### Delphi consensus statements

A total of 82 statements were generated. The Delphi consensus was concluded after three rounds at the end of September 2023 with a majority (92.7% [76/82]) of the statements reaching an agreement.

Supplement 4 shows the establishment of the key aspects and statements over the three Delphi rounds.

**Figure 2:**
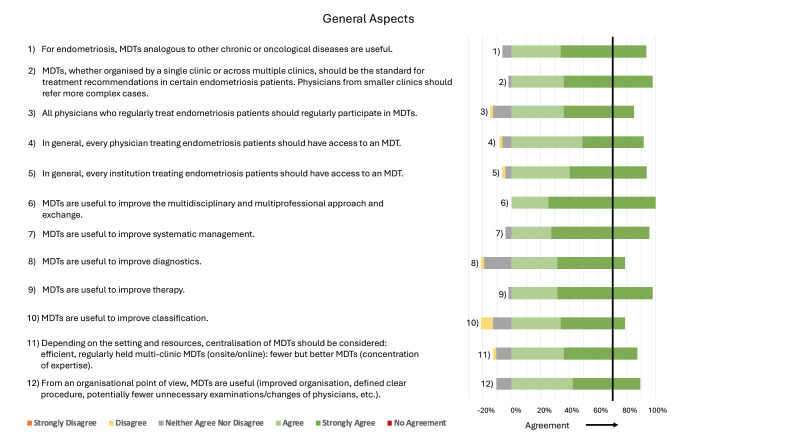
Consensus statements regarding General Aspects. Consensus was reached with ≥ 70% Agree/Strongly Agree. For those statements for which no consensus was reached, the deficit is indicated by a red bar up to the 70% consensus threshold. On the left side of the 0 axis are the percentages of strongly disagree, disagree, neither agree nor disagree, and on the right, agree and strongly agree are shown. In general, 47 respondents answered all consensus statements, and in the few cases where this number differs, it is displayed directly after the statement (n=).

**Figure 3:**
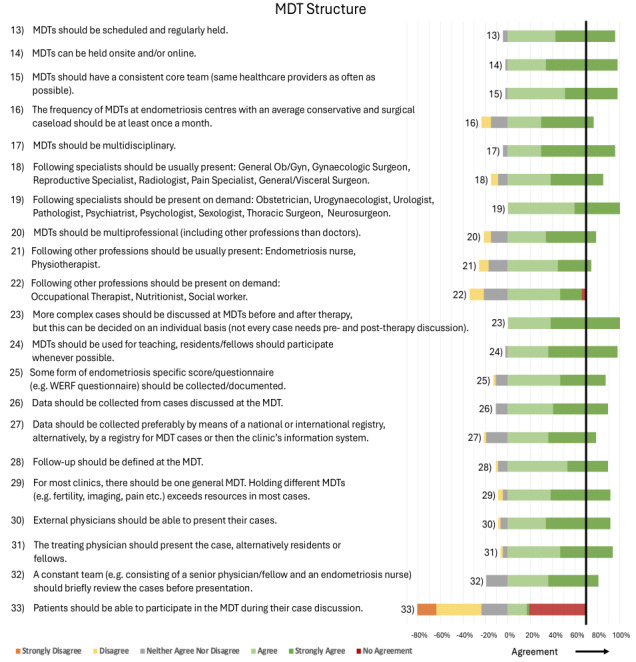
Consensus statements regarding MDT Structure.

**Figure 4:**
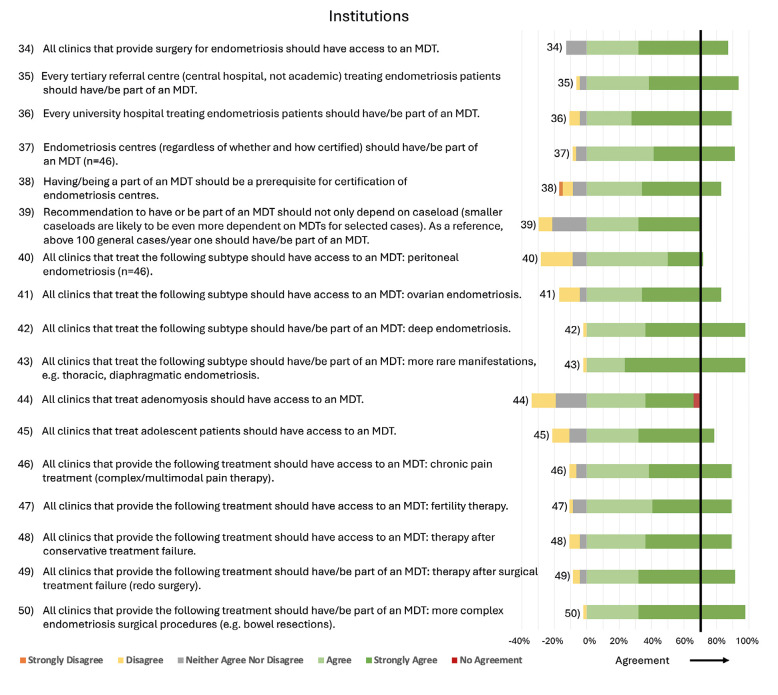
Consensus statements regarding Institutions.

**Figure 5:**
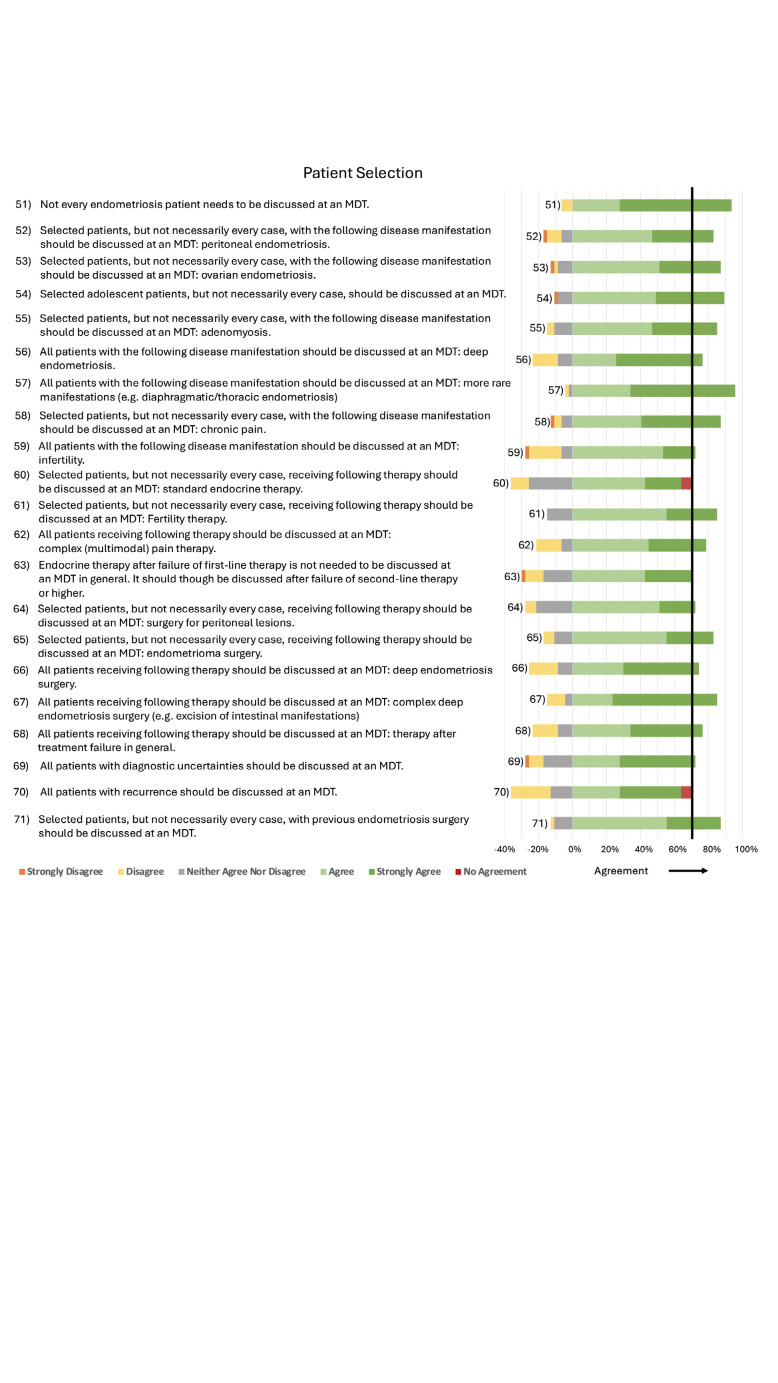
Consensus statements regarding Patient Selection.

**Figure 6:**
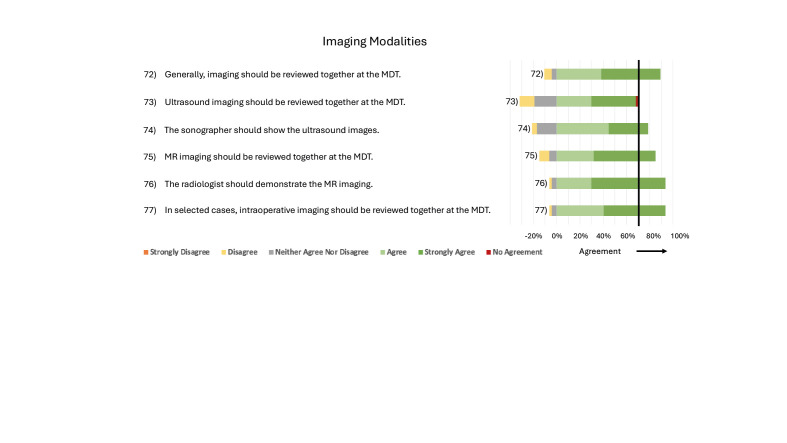
Consensus statements regarding Imaging Modalities.

**Figure 7:**
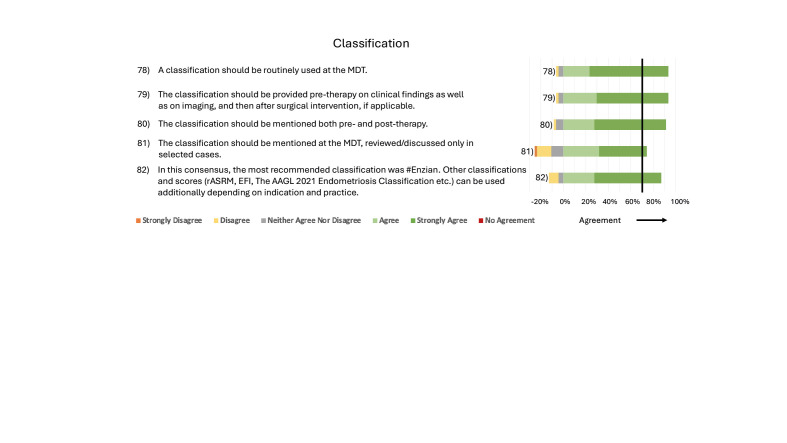
Consensus statements regarding Classification.

**Figure 8:**
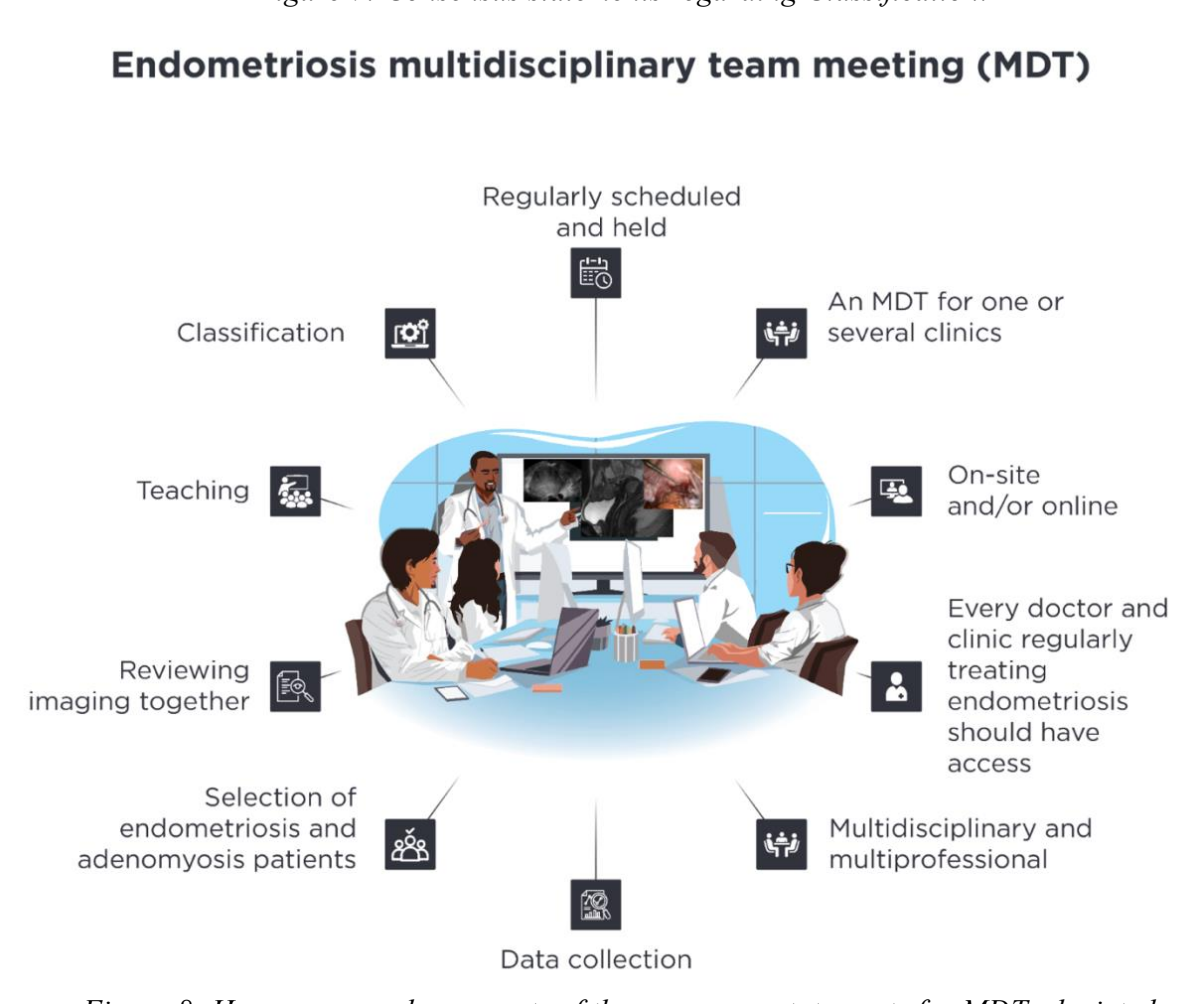
Here are some key aspects of the consensus statements for MDTs depicted.

Detailed statements and their agreement rates can be found in Figures 2–7. A selection of key aspects of the consensus statements for MDTs can be found depicted in Figure 8.

The following section highlights some key aspects of the statements, categorised into the above mentioned topics.

#### General Aspects

It is recommended that all physicians and institutions who regularly treat endometriosis patients have access to MDTs and participate in them regularly. MDTs are beneficial for improving multidisciplinarity and multiprofessional collaboration. They serve to optimise systematic management of the disease, including classification, clinical and imaging diagnosis and medical, complementary and surgical therapy. The centralisation of MDTs covering multiple centres should be considered to consolidate expertise and enhance efficiency (Figure 2).

#### MDT Structure

MDTs should be scheduled and held regularly, either onsite and/or online. MDTs should maintain a consistent core team. The recommended frequency for MDTs is at least once a month. The MDTs should be multidisciplinary with specific medical specialties forming the core team (usually present) and other specialties available on demand for specific cases. MDTs should also be multiprofessional and include non-physician healthcare professionals. Selected cases should be discussed with the MDT before and after therapy, although this process can be determined on an individual basis. Data should be gathered from cases discussed at the MDT, and follow-up plans should be defined during MDTs. A form of endometriosis specific questionnaire should be collected and documented. External physicians should have the opportunity to present their cases, and MDTs should serve as a teaching tool for residents and the involved team.

The respondents opposed the idea of allowing patients to participate in their own case discussions (Figure 3).

#### Institutions

Every tertiary referral centre treating endometriosis patients, every university hospital treating endometriosis patients, and every endometriosis centre (regardless of whether and how it was certified) should be part of an MDT. Being part of an MDT should be a prerequisite for the certification of endometriosis centres. All institutions that treat adolescent patients should have access to an MDT. The respondents have also defined for which therapeutic services a centre should have an MDT (have/be part of an MDT) and for which therapies access to an MDT should be available if needed (have access to an MDT).

The statement ‘All institutions that treat adenomyosis should have access to an MDT’ did not reach an agreement (Figure 4).

#### Patient Selection

Not every endometriosis patient needs to be discussed at an MDT. The respondents have defined which patients with which disease manifestation should be presented at the MDT (Figure 5).

#### Imaging Modalities

Imaging should be reviewed at MDTs. This process should also include MRIs presented by a radiologist. In selected cases, intraoperative imaging should be presented at MDTs. No agreement was reached for the general viewing of ultrasound imaging in all cases. However, if ultrasound imaging is included, it should be presented by the examining sonographer (Figure 6).

#### Classification

A classification system should be routinely used at the MDT. The classification system, if applicable, should be mentioned both in the pre- and post- therapeutic settings. The presented classification should be reviewed/discussed only in selected cases. In this consensus, the most recommended classification was the #Enzian classification (Keckstein et al., 2021). Additional classifications and scores may be used as needed depending on indication and practice (Figure 7).

## Discussion

This study presents the first analysis of the current status and recommendations for MDTs for endometriosis and adenomyosis, with the consensus group agreeing on most (92.7%, 76) of the 82 statements. Achievement of the high level of agreement in an international consensus group that consisted of participants from 21 countries and included institutions ranging from private practices to university hospitals from various healthcare systems underscores the significance and broad applicability of our recommendations. The majority agreed that MDTs are useful thus recognising the MDT-associated benefits, which align with those reported in the available literature (Ugwumadu et al., 2017; Bolze et al., 2019; Allaire et al., 2020).

The existing MDTs are multidisciplinary in 97% of the cases and multiprofessional in 63.6%, both of which agree with the consensus statements for implementation and conduct of MDTs. This implementation appears to enhance standardised patient care and reduce questionable practices (Ugwumadu et al., 2017; Haward et al., 2003; Wagner, 2004; Mickan, 2005) and might help increase awareness on the different levels that are involved in terms of healthcare providers and institutions thus facilitating patient referrals to adequate centres. This process might allow for early diagnosis, especially in case of deep infiltration and involvement of the uterus and other organs (Ghai et al., 2020). MDTs seem to be especially promising for complex situations in which clinical decisions extend existing guidelines. Thoughtful patient selection seems crucial for ensuring the benefits and relevance of MDTs. The lead role during an MDT should be assumed by the gynaecologist guiding the patient through the diagnosis and treatment.

The respondents agreed that MDTs are beneficial for teaching. Furthermore, the MDTs have the potential to educate the entire team concerning all aspects of diagnosis and treatment, including a professional multidisciplinary evaluation of imaging results by comparing clinical symptoms and findings with ultrasound and MRI images and surgical results. Thus, a collaborative imaging review at the MDT, especially of MRI scans, was recommended by the respondents. The recommendations for reviewing all ultrasound findings narrowly missed the agreement threshold despite consideration of transvaginal ultrasound (TVUS) as the primary diagnostic method due to its accessibility and cost-effectiveness (Condous et al., 2024). A possible reason could be the fact that ultrasound images are not widely available in digital format. In our opinion, digital storage of standardised, high-quality, and ideally pre- classified images in the institution’s information system should be considered an important part of the implementation of an adequate diagnostic approach for each centre. Another explanation could be the high dependence of ultrasound imaging on the examiners’ experience in terms of producing reproducible images and videos compared to the static MRI modality. Practices in TVUS imaging vary widely between countries and centres; however, regardless of whether a surgeon- sonographer, a radiologist, or a sonographer obtains the TVUS, the same examiner should present the images and findings if such findings are discussed during an MDT. Ideally, a pre- and post-therapeutic classification in imaging and surgery should be used to communicate in the same scientific language when discussing endometriosis and adenomyosis cases. The recent developments in pre-therapeutic imaging and its impact on therapy planning represent milestones in the treatment of patients (Condous et al., 2024; Keckstein et al., 2023; Thomassin-Naggara et al., 2020).

While the survey addressing existing MDTs showed the use of different classifications (Table II), the respondents agreed predominantly on the use of the #Enzian classification for implementation and conduct of MDTs and agreed that additional or alternative classification systems could be used based on specific indications and practices. Considering the implications of such a system, no doubts exist as to the advantages of advocating for a unified classification system for future applications.

It is not clear why the statement that all centres treating adenomyosis should have access to an MDT did not reach agreement among the respondents. This finding contradicts the statements that every physician and institution involved in the treatment of endometriosis should have access to an MDT and that selected adenomyosis cases should be presented at an MDT. In our opinion, the possible concurrent presence of adenomyosis should be considered in all endometriosis patients, and treatment options in symptomatic and/or infertile patients should be added to the treatment regimen (Sharara et al., 2021; Guo, 2023). The fact that adenomyosis lags behind endometriosis in terms of attention and knowledge may have influenced the respondents’ decisions (Munro, 2021).

A patient-centred individualised approach that considers several factors, such as age, family planning status, symptoms, and clinical history, including previous endometriosis-related treatments, represents an accepted modern concept in endometriosis care. However, the idea that patients should participate in their own case discussions was clearly rejected by the respondents. Instead of direct participation, the final MDT recommendation should be discussed with the patient after the MDT and include detailed informed consent regarding medical and surgical treatments.

MDTs primarily focus on the disease and its management rather than the associated complications. The agreement on presenting cases at MDTs after surgical/general treatment failure shows the importance of addressing unexpected outcomes and complications during MDTs, especially as complication rates in complex endometriosis surgeries are significant (Hudelist et al., 2022). Inclusion of the presentation and discussion of complications as part of MDTs could be beneficial in terms of quality control.

In contrast to the advantages of MDT implementation, incorporartion of quality control might also be related to certain challenges (Ugwumadu et al., 2017), which include time restraints, administrative and logistical burdens, limited resources, varying caseloads, and costs. In this context, the consensus group recommends centralisation of MDTs and networking. This process could help streamline administrative services and resources to the areas in which such services are needed. Smaller centres could present their patients at a central MDT. Virtual platforms would be an option for facilitating communication and resource utilisation. Cooperative networks between different centres could distribute workload and could also allow for multicentre research with centralised digital data collection tools and multicentre scientific working groups. Almost 90% of respondents agreed on the recommendation that collecting data of cases presented in MDTs would be beneficial. This step could enhance quality assurance through various databases and benchmark studies similar to those done in other surgical domains (Gero et al., 2019; Wu et al., 2023; Khalil et al., 2021).

Limitations of this work include the limited scientific foundation concerning MDTs for endometriosis and adenomyosis. Additionally, this study relied on an online survey for data collection. Anonymity facilitated free expression, but some opinions may have been influenced by negative experiences and unfamiliarity with MDTs. The recommendations presented here are not conclusive; rather, they aim to facilitate the establishment and development of discourse concerning MDTs. Future scientific evaluations of the MDT process should be conducted.

## Conclusion

In this study, we assessed the current status of endometriosis MDTs and used a Delphi consensus method to develop 76 recommendations for implementation and conduct. Our findings demonstrate that the consensus group recognises the benefits of endometriosis MDTs. MDTs play an important role in establishing a basis for guideline- driven, multidisciplinary, and individualised care by effectively addressing the intricate and ongoing challenges associated with endometriosis and adenoymosis.

## Supplementary material

Supplement I

Supplement II

Supplement III

Supplement IV
