# Supplementary material for: Recommendations for the implementation and conduct of multidisciplinary team meetings for those providing endometriosis and adenomyosis care - a Delphi consensus of the European Endometriosis League (EEL)
Source: Facts Views Vis Obgyn. 2024 Sep 30;16(3):337–50. doi: 10.52054/FVVO.16.3.038 (PMC11569442; doi:10.52054/FVVO.16.3.038)
Supplement: Supplement I [file FVVinObGyn-16-337-s001.pdf]

- *The respondents' data and expertise were collected separately.*
- *Comments and suggestions for corrections could be provided for each question and at the end of the questionnaire by the respondents*

1. How relevant do you consider an MDT for improving endometriosis management (in selected cases)?
   - not relevant
   - barely relevant
   - moderately relevant
   - relevant
   - very relevant

2. Should every institution treating endometriosis patients have an MDT nowadays (or access to an MDT for special cases)?
   - Yes
   - No

3. Should it be mandatory for certified endometriosis centres to have an MDT?
   - Yes
   - No

4. Approximately how many patients with endometriosis does your institution treat per year (outpatient and inpatient, conservative and surgical cases)?
   - 0-100
   - 101-250
   - 251-500
   - 501-1000
   - >1000

5. Approximately how many patients with endometriosis are operated at your institution per year?
   - <50
   - 50-100
   - 101-250
   - 251-500
   - >500

6. How many patients with endometriosis that you treat (outpatient and inpatient, conservative and surgical cases) at your institution per year would you describe as complex cases (e.g., deep endometriosis)?
   - <50
   - 50-100
   - 101-250
   - >250

7. Does your work site already has an MDT?
   - Yes
   - No

Survey of existing MDTs:

8. Approximately what percentage of cases are discussed at the MDT compared to the total volume?
   - Less than 25%
   - approx. 25-50%
   - approx. 50-75%
   - approx. 75-100%

9. How often does the MDT meet?
   - More than once per week
   - Once per week
   - One per 2 weeks
   - Once per month
   - Less than once per month

10. What cases are discussed?
    - All cases
    - Only complicated
    - Only deep endometriosis

11. Who can register, only own cases or also external cases from e.g. other clinics or physicians in private practice?
    - Only own cases
    - Also other

12. Who presents the cases?
    - the registering doctors
    - all cases are always presented by the same doctor in charge

13. Would you describe your MDT as multidisciplinary (Are other medical disciplines present, e.g. radiologists, urologists, surgeons)?
    - Yes
    - No

14. Besides the gynaecologists treating endometriosis, are other subspecialties of gynaecology present, e.g., reproductive specialists)?
    - Yes
    - No

15. Would you describe your MDT as multiprofessional (are other professions present besides medical specialties, e.g., physiotherapists, social workers, endometriosis nurses)?
    - Yes
    - No

16. Who usually participates, which professions are usually represented?
    - Gynaecologic Surgeon
    - Reproductive Specialist
    - Obstetrician
    - Radiologist

- General/Visceral Surgeon
- Urologist
- Pathologist
- Pain specialist
- Endometriosis Nurse
- Nutritionist
- Physiotherapist
- Social Worker
- Other (please specify)

17. How important do you think it is to review imaging together (Ultrasound/MRI/Intraoperative imaging)?
    - not relevant
    - barely relevant
    - moderately relevant
    - relevant
    - very relevant

18. Is ultrasound imaging shown at the MDT?
    - Yes
    - No

19. Is MRI imaging shown at the MDT?
    - Yes
    - No

20. Is MRI imaging shown by a radiologist at the MDT?
    - Yes
    - No

21. Is intraoperative imaging (e.g. laparoscopic videos) shown at the MDT?
    - Yes always
    - Yes, special cases
    - No

22. Is the sonographer also the surgeon in your institution?
    - In most cases yes
    - In most cases no

23. Which classification/s is/are used?
    - rASRM
    - #Enzian
    - EFI
    - The AAGL 2021 Endometriosis Classification
    - Other (please specify)

24. Is the classification given pre- and post-therapy?
    - Post-therapy only
    - Both

25. Are cases discussed pre- and post-therapy whenever possible?
    - Only pre-therapy
    - Only post-therapy
    - Both

26. Who determines classification?
    - The enrolling gynaecologist
    - It is determined or changed at the MDT
    - Other, enter a comment if needed

27. Are more conservative or surgical cases discussed?
    - Mainly operative
    - Mainly conservative
    - Balanced

28. Is some form of follow-up defined?
    - Yes
    - No

29. Is there any form of data collection at the MDT?
    - Yes
    - No

30. You think an MDT is helpful for teaching of the younger colleagues?
    - Yes
    - No

The following questions were for all respondents whether or not an MDT exists. The following aspects about MDTs were rated using a 5-point Likert scale from "not relevant" to "very relevant", indicating whether they should be revisited in the next round.

31. How important do you think it is to determine which hospitals should have an MDT (size, centre, academic, etc.)

32. MDT frequency

33. Implementation of MDTs depending on case load

34. What cases should be discussed

35. Whether patients should be presented pre- and post-therapy

36. Which professions/disciplines should participate

37. Who should present the cases

38. What imaging should be shown

39. Which classifications should be provided

40. Which follow-up should be discussed

41. What kind of data collection should take place

42. Teaching at the MDT
