# Supplementary material for: Recommendations for the implementation and conduct of multidisciplinary team meetings for those providing endometriosis and adenomyosis care - a Delphi consensus of the European Endometriosis League (EEL)
Source: Facts Views Vis Obgyn. 2024 Sep 30;16(3):337–50. doi: 10.52054/FVVO.16.3.038 (PMC11569442; doi:10.52054/FVVO.16.3.038)
Supplement: Supplement II [file FVVinObGyn-16-337-s002.pdf]

- *Comments and suggestions for corrections could be provided for each question and at the end of the questionnaire by the respondents*

General Aspects and MDT Structure

1. How often should an MDT be recommended to an endometriosis centre with an average caseload of several conservative and surgical endometriosis therapies per week?
- More than once a week
- Once a week
- Once every two weeks
- Once a month
- Less than once a month
- Let each clinic decide for itself

2. Should an MDT be multidisciplinary?
- Yes
- No

3. If yes, which medical specialties are recommended to be present as the "core team" (usually present)?
- General Ob/Gyn
- Surgeon Gynaecologist
- Reproductive Specialist
- Obstetrician
- Urogynaecologist
- Radiologist
- General/Visceral Surgeon
- Urologist
- Pathologist
- Pain Specialist
- Psychiatrist / Psychologist
- Sexologist

4. Which medical specialties are recommended to be present depending on the question (present if needed, please only mention if you did not mention above already)?
- General Ob/Gyn
- Gynaecologic Surgeon
- Reproductive Specialist
- Obstetrician
- Urogynaecologist
- Radiologist
- General/Visceral Surgeon
- Urologist
- Pathologist
- Pain Specialist
- Psychiatrist / Psychologist
- Sexologist
- Thoracic Surgeon

- Neurosurgeon

5. Should an MDT be multiprofessional?
- Yes
- No

6. If yes, which other professions are recommended to be present as the "core team" (usually present)?
- Endometriosis nurse
- Nutritionist
- Physiotherapist
- Occupational Therapist
- Social Worker

7. Which other professions are recommended to be present depending on the question (present if needed, please only mention if you did not mention above already)?
- Endometriosis nurses
- Nutritionist
- Physiotherapist
- Occupational Therapist
- Social Worker

8. Should the cases be presented whenever possible pre- and post-therapy?
- Yes
- No, only pre-therapy
- Not relevant, decision depending on the individual case

9. Should the MDT be used for teaching, should residents participate whenever possible?
- Yes
- No

10. Should some form of quality-of-life score/questionnaire (endometriosis specific e.g. WERF Questionnaire or then non-specific e.g. SF-36 Questionnaire) be collected/documented (analogous to tumour MDTs e.g. ECOG Status)?
- Yes, any form of questionnaire
- Yes, an endometriosis specific questionnaire
- Yes, a non-specific questionnaire
- No

11. Should data be collected from cases discussed at the MDT?
- Yes
- No

12. If data collection should be recommended, how?
- Separate special registry for MDT cases (own clinic registry)
- National or international registry (if available)
- Clinic information system

13. Should a follow-up be recommended at the MDT?
- Yes
- No

14. In what mode should an MDT take place?
- On site only
- On site and online if needed for external physicians, multi-clinic MDTs

15. In general, would it make more sense, if possible, to have efficient multi-clinic MDTs (online, centralisation of costs, administration) (less MDTs but better ones)?
- Yes
- No

16. From an organisational and financial point of view: do endometriosis MDTs make sense from your point of view (better organisation, fewer unnecessary examinations, change of doctors, etc.)?
- Yes
- No

17. If the caseload and the organisational capacity exist, does it make sense to hold different MDTs (e.g. fertility, imaging, pain...etc.)?
- Yes
- Yes, depending on the case load
- No

18. Should external physicians be able to present their cases?
- Yes
- No not directly, they should refer patients through the consultation and then they are presented by the clinical team

19. Who should present the cases (if someone from the clinic presents it)?
- Resident doctor
- Senior physician or Fellow
- the physician in charge of the case
- Doesn't matter

20. Is it useful to have a team, e.g. consisting of a senior physician and an endometriosis nurse if available, who prepare and briefly review the cases before presentation?
- Yes
- No

Institutions

- *For the following questions, respondents could choose from the following options:*

• *Yes*
• *No, but they should have access to an MDT*
• *No*

Alternatively, they could provide a comment or suggest a correction.

21. Should all practitioners treating endometriosis patients in general have an MDT at their institution?
22. Should all hospitals that operate on endometriosis patients in general have their own MDT?
23. Should every certified endometriosis centre have its own MDT, respectively should an MDT be a prerequisite for certification?
24. Should every tertiary referral centre (central hospital, not academic) treating endometriosis patients be recommended to have its own MDT?
25. Should every university hospital treating endometriosis patients be recommended to have its own MDT?
26. At what caseload per year should an MDT be recommended in a clinic (generally all cases) (multi-clinic MDT are also included here)?
27. Should an MDT be recommended for all clinics that treat this subtype: peritoneal endometriosis?
28. Should an MDT be recommended for all clinics that treat this subtype: ovarian endometriosis (OMA)?
29. Should an MDT be recommended for all clinics that treat this subtype: deep endometriosis?
30. Should an MDT be recommended for all clinics that treat this subtype: more rare manifestations, e.g. thoracic, diaphragmatic endometriosis?
31. Should an MDT be recommended for all clinics that treat adolescent patients?
32. Should an MDT be recommended for all clinics providing this therapy: chronic pain treatment (complex pain therapy)?
33. Should an MDT be recommended for all clinics providing this therapy: fertility therapy?
34. Should an MDT be recommended for all clinics providing therapy after conservative treatment failure?
35. Should an MDT be recommended for all clinics providing therapy after surgical treatment failure (redo surgery)?
36. Should all clinics which perform more complex endometriosis surgical procedures, e.g. bowel resections have their own MDT?

Patient Selection

- *For the following questions, respondents could choose from the following options:*

- *Yes*
- *No, only selected cases*
- *No*

Alternatively, they could provide a comment or suggest a correction.

37. Should it be recommended that every endometriosis patient be discussed at an MDT, regardless of disease severity and treatment modality?

38. Should an MDT be recommended for the following manifestation in general: peritoneal endometriosis?

39. Should an MDT be recommended for the following manifestation in general: deep endometriosis?

40. Should an MDT be recommended for the following manifestation in general: ovarian endometriosis (OMA)?

41. Should an MDT be recommended for the following manifestation in general: more rare manifestations, e.g. diaphragmatic, thoracic endometriosis?

42. Should an MDT be recommended for chronic (e.g. pelvic) pain patients in general?

43. Should an MDT be recommended for adolescent patients in general?

44. Should an MDT be recommended for endometriosis patients with infertility?

45. Should an MDT be recommended for the following therapy in general: Fertility therapy?

46. Should an MDT be recommended for the following therapy in general: complex pain therapy?

47. Should an MDT be recommended for the following therapy in general: standard endocrine therapy?

48. Should an MDT be recommended for the following therapy in general: Endocrine therapy after failure of basic therapy (failure of first-line therapy)?

49. Should an MDT be recommended for the following therapy in general: peritoneal excision?

50. Should an MDT be recommended for the following therapy in general: endometrioma surgery?

51. Should an MDT be recommended for the following therapy in general: deep endometriosis surgery?

52. Should an MDT be recommended for the following therapy: complex deep endometriosis surgery, e.g. excision of intestinal manifestations?

53. Should an MDT be recommended for cases of treatment failure in general?

54. Should an MDT be recommended for cases of diagnostic uncertainties in general?

55. Should an MDT be recommended for cases of recurrence (symptoms, disease manifestation) in general?

56. Should an MDT be recommended for cases with previous endometriosis surgery?

Imaging Modalities

57. In general, should imaging at the MDT be re-viewed together?
- Yes
- In selected cases
- No

58. Should ultrasound imaging at the MDT be re-viewed together?
- Yes
- In selected cases
- No

59. Who should show the ultrasound imaging?
- The person who did the ultrasound whenever possible
- The presenting doctor
- Not relevant

60. Should MR imaging at the MDT be re-viewed together (if available)?
- Yes
- In selected cases
- No

61. Who should show the MR imaging?
- Radiologist
- The presenting doctor
- not relevant

62. Should intraoperative imaging at the MDT be re-viewed together?
- Yes
- In selected cases
- No

Classification

63. Which classification/s should be recommended to use?
- rASRM
- #Enzian
- EFI
- The AAGL 2021 Endometriosis Classification
- Other

64. Should the classification always be briefly reviewed/discussed again at the MDT?
- Yes
- Only selected cases
- No

65. Should the classification at the MDT be provided pre- and post-therapeutically?
- Post-therapy only
- Pre-therapy only
- Both
