# Supplementary material for: Recommendations for the implementation and conduct of multidisciplinary team meetings for those providing endometriosis and adenomyosis care - a Delphi consensus of the European Endometriosis League (EEL)
Source: Facts Views Vis Obgyn. 2024 Sep 30;16(3):337–50. doi: 10.52054/FVVO.16.3.038 (PMC11569442; doi:10.52054/FVVO.16.3.038)
Supplement: Supplement III [file FVVinObGyn-16-337-s003.pdf]

- *Comments and suggestions for corrections could be provided for each question and at the end of the questionnaire by the respondents*
- *For each of the following statements, respondents could indicate their agreement or disagreement using a 5-point Likert scale (strongly disagree, disagree, neither agree nor disagree, agree, strongly agree)*

*General Aspects*

1. For endometriosis, MDTs analogous to other chronic diseases or tumour MDTs are useful.

2. MDTs, whether organised by a single clinic or multi-centred organised should be the standard for treatment recommendations in a part of endometriosis patients. Physicians from smaller clinics or medical practices should refer to them for more complex cases.

3. All physicians who regularly treat endometriosis patients should regularly participate in MDTs.

4. In general, every physician treating endometriosis patients should have access to an MDT.

5. In general, every institution treating endometriosis patients should have access to an MDT.

6. MDTs are useful to improve the interdisciplinary and interprofessional approach and exchange about the disease.

7. MDTs are useful to improve systematic management of the disease.

8. MDTs are useful to improve diagnostics of endometriosis.

9. MDTs are useful to improve the therapy of endometriosis.

10. MDTs are useful to improve the classification of endometriosis.

11. Depending on the setting and resources, centralisation of MDTs should be considered: efficient, regularly held multicentre MDTs (onsite/online): fewer but better MDTs (concentration of expertise).

12. From an organisational point of view, MDTs are useful (improved organisation, defined clear procedure, potentially fewer unnecessary examinations/changes of physicians, etc.).

*MDT Structure*

13. MDTs should be scheduled and regularly held.

14. MDTs can be held onsite and/or online.
15. MDTs should have a consistent core team (more or less the same health care providers as often as possible).
16. The frequency of MDTs at endometriosis centres with an average conservative and surgical caseload should be at least once a month.
17. MDTs should be multidisciplinary.
18. Following specialists should be usually present: General Ob/Gyn, Gynaecologic Surgeon, Reproductive Specialist, Radiologist, Pain Specialist, General/Visceral Surgeon.
19. Following specialists should be present on demand: Obstetrician, Urogynaecologist, Urologist, Pathologist, Psychiatrist, Psychologist, Sexologist, Thoracic Surgeon, Neurosurgeon.
20. MDTs should be multiprofessional (including other professions than doctors).
21. Following other professions should be usually present: Endometriosis nurse, Physiotherapist.
22. Following other professions should be present on demand: Occupational Therapist, Nutritionist, Social worker.
23. More complex cases should be discussed at MDTs before and after therapy, but this can be decided on an individual basis (not every case needs pre- and post-therapy discussion).
24. MDTs should be used for teaching, residents/fellows should participate whenever possible.
25. Some form of endometriosis specific quality of life score/questionnaire (endometriosis specific e.g. WERF Questionnaire) should be collected/documented.
26. Data should be collected from cases discussed at the MDT.
27. Data should be collected preferably by means of a national or international registry, alternatively by a registry for board cases or then the clinic's information system.
28. Follow-up should be defined at the MDT.
29. For most clinics, there should be one general MDT. Holding different MDTs (e.g. fertility, imaging, pain etc.) exceeds resources in most cases.
30. External physicians should be able to present their cases.
31. The treating physician should present the case, alternatively residents or fellows.

32. A constant team (e.g. consisting of a senior physician/fellow and an endometriosis nurse) should briefly review the cases before presentation.

33. Patients should be able to participate in the MDT during their case discussion.

*Institutions*

34. All clinics that provide surgery for endometriosis should have access to an MDT.

35. Every tertiary referral centre (central hospital, not academic) treating endometriosis patients should have/be part of an MDT.

36. Every university hospital treating endometriosis patients should have/be part of an MDT.

37. Endometriosis centres (regardless of whether and how certified) should have/be part of an MDT.

38. Having/being a part of an MDT should be a prerequisite for certification of endometriosis centres.

39. Recommendation to have or be part of an MDT should not only depend on caseload (smaller caseloads are likely to be even more dependent on MDTs for selected cases). As a reference, above 100 general cases/year one should have/be part of an MDT.

40. All clinics that treat the following subtype should have access to an MDT: peritoneal endometriosis.

41. All clinics that treat the following subtype should have access to an MDT: ovarian endometriosis.

42. All clinics that treat the following subtype should have/be part of an MDT: deep endometriosis.

43. All clinics that treat the following subtype should have/be part of an MDT: more rare manifestations, e.g. thoracic, diaphragmatic endometriosis.

44. All clinics that treat adenomyosis should have access to an MDT.

45. All clinics that treat adolescent patients should have access to an MDT.

46. All clinics that provide the following treatment should have access to an MDT: chronic pain treatment (complex/multimodal pain therapy).

47. All clinics that provide the following treatment should have access to an MDT: fertility therapy.

48. All clinics that provide the following treatment should have access to an MDT: therapy after conservative treatment failure.

49. All clinics that provide the following treatment should have/be part of an MDT: therapy after surgical treatment failure (redo surgery).

50. All clinics that provide the following treatment should have/be part of an MDT: more complex endometriosis surgical procedures (e.g. bowel resections).

*Patient Selection*

51. Not every endometriosis patient needs to be discussed at an MDT.

52. Selected patients, but not necessarily every case, with the following disease manifestation should be discussed at an MDT: peritoneal endometriosis.

53. Selected patients, but not necessarily every case, with the following disease manifestation should be discussed at an MDT: ovarian endometriosis.

54. Selected adolescent patients, but not necessarily every case, should be discussed at an MDT.

55. Selected patients, but not necessarily every case, with the following disease manifestation should be discussed at an MDT: adenomyosis.

56. All patients with the following disease manifestation should be discussed at an MDT: deep endometriosis.

57. All patients with the following disease manifestation should be discussed at an MDT: more rare manifestations (e.g. diaphragmatic/thoracic endometriosis)

58. Selected patients, but not necessarily every case, with the following disease manifestation should be discussed at an MDT: chronic pain.

59. All patients with the following disease manifestation should be discussed at an MDT: infertility.

60. Selected patients, but not necessarily every case, receiving following therapy should be discussed at an MDT: standard endocrine therapy.

61. Selected patients, but not necessarily every case, receiving following therapy should be discussed at an MDT: Fertility therapy.

62. All patients receiving following therapy should be discussed at an MDT: complex (multimodal) pain therapy.

63. Endocrine therapy after failure of first-line therapy is not needed to be discussed at an MDT in general. It should though be discussed after failure of second-line therapy or higher.

64. Selected patients, but not necessarily every case, receiving following therapy should be discussed at an MDT: surgery for peritoneal lesions.

65. Selected patients, but not necessarily every case, receiving following therapy should be discussed at an MDT: endometrioma surgery.

66. All patients receiving following therapy should be discussed at an MDT: deep endometriosis surgery.

67. All patients receiving following therapy should be discussed at an MDT: complex deep endometriosis surgery (e.g. excision of intestinal manifestations)

68. All patients receiving following therapy should be discussed at an MDT: therapy after treatment failure in general.

69. All patients with diagnostic uncertainties should be discussed at an MDT.

70. All patients with recurrence should be discussed at an MDT.

71. Selected patients, but not necessarily every case, with previous endometriosis surgery should be discussed at an MDT.

*Imaging Modalities*

72. Generally, imaging should be re-viewed together at the MDT.

73. Ultrasound imaging should be re-viewed together at the MDT.

74. The sonographer should show the ultrasound images.

75. MR imaging should be re-viewed together at the MDT.

76. The radiologist should demonstrate the MR imaging.

77. In selected cases, intraoperative imaging should be re-viewed together at the MDT.

*Classification*

78. A classification should be routinely used at the MDT.

79. The classification should be provided pre-therapy on clinical findings as well as on imaging, and then after surgical intervention, if applicable.

80. The classification should be mentioned pre- and post-therapeutically.

81. The classification should be mentioned at the MDT, reviewed/discussed only in selected cases.

82. In this consensus, the most recommended classification was #Enzian. Other classifications and scores (rASRM, EFI, The AAGL 2021 Endometriosis Classification etc.) can be used additionally depending on indication and practice.
