# Supplementary material for: Recommendations for the implementation and conduct of multidisciplinary team meetings for those providing endometriosis and adenomyosis care - a Delphi consensus of the European Endometriosis League (EEL)
Source: Facts Views Vis Obgyn. 2024 Sep 30;16(3):337–50. doi: 10.52054/FVVO.16.3.038 (PMC11569442; doi:10.52054/FVVO.16.3.038)
Supplement: Supplement IV [file FVVinObGyn-16-337-s004.pdf]

Topics	*Round 1*	*Round 2*	*Round 3*
General Aspects	1. In selected cases, MDTs are relevant for improving endometriosis management<br><br>2. Every institution treating endometriosis patients should have access to an MDT for selected cases<br><br>3. Cooperation between small and large clinics for MDTs	1. In selected cases, MDTs are relevant for improving endometriosis management<br><br>2. All practitioners treating endometriosis patients should have access to an MDT<br><br>3. Every institution treating endometriosis patients should have access to an MDT for selected cases<br><br>4. Cooperation between small and large clinics for MDTs<br><br>5. Efficient multi-clinic MDTs are meaningful, centralisation of costs, administration)	1. For endometriosis, MDTs analogous to other chronic or oncological diseases are useful.<br><br>2. MDTs, whether organised by a single clinic or across multiple clinics, should be the standard for treatment recommendations in certain endometriosis patients. Physicians from smaller clinics should refer more complex cases.<br><br>3. All physicians who regularly treat endometriosis patients should regularly participate in MDTs.<br><br>4. In general, every physician treating endometriosis patients should have access to an MDT.<br><br>5. In general, every institution treating endometriosis patients should have access to an MDT.<br><br>6. MDTs are useful to improve the multidisciplinary

and multiprofessional approach and exchange.

7. MDTs are useful to improve systematic management.

8. MDTs are useful to improve diagnostics.

9. MDTs are useful to improve therapy.

10. MDTs are useful to improve classification.

11. Depending on the setting and resources, centralisation of MDTs should be considered: efficient, regularly held multi-clinic MDTs (onsite/online): fewer but better MDTs (concentration of expertise).

12. From an organisational point of view, MDTs are useful (improved organisation, defined clear procedure, potentially fewer unnecessary examinations/changes of physicians, etc.).

MDT Structure	4. MDT frequency<br>5. MDT regularly scheduled<br>6. One MDT per centre or for multiple centres<br>7. MDT onsite and/or online<br>8. A general endometriosis MDT or different ones (e.g. Fertility, Imaging, Pain)?<br>9. Implementation of MDTs depending on case load<br>10. Should patients be presented pre- and post-therapy?<br>11. Multidisciplinary MDTs<br>12. Multiprofessional MDTs<br>13. Which health care professions should participate<br>14. Presenter of cases<br>15. External Physicians	6. MDT recommended to an endometriosis centre with an average caseload: 1x/month<br>7. MDT regularly scheduled<br>8. MDT onsite and/or online<br>9. MDT should be multidisciplinary<br>10. MDT core Team: General Ob/Gyn, Gynaecologic Surgeon, Reproductive Specialist, Radiologist, Pain specialist, General/Visceral Surgeon<br>11. MDT Team on demand: Obstetrician, Urogynaecologist, Urologist, Pathologist, Psychiatrist, Psychologist, Sexologist, Thoracic Surgeon, Neurosurgeon<br>12. MDTs should be multiprofessional (including other professions than doctors)<br>13. Other professions MDT core team: Endometriosis nurse, Physiotherapist	13. MDTs should be scheduled and regularly held.<br>14. MDTs can be held onsite and/or online.<br>15. MDTs should have a consistent core team (same healthcare providers as often as possible).<br>16. The frequency of MDTs at endometriosis centres with an average conservative and surgical caseload should be at least once a month.<br>17. MDTs should be multidisciplinary.<br>18. Following specialists should be usually present: General Ob/Gyn, Gynaecologic Surgeon, Reproductive Specialist, Radiologist, Pain Specialist, General/Visceral Surgeon.<br>19. Following specialists should be present on demand: Obstetrician, Urogynaecologist, Urologist, Pathologist, Psychiatrist,

	can present own cases<br><br>16. Follow-up<br><br>17. Data collection<br><br>18. Teaching, Trainees should attend	14. Other professions MDT on demand: Occupational Therapist, Nutritionist, Social worker<br><br>15. Selected cases MDT discussion pre- and after therapy<br><br>16. MDTs should be used for teaching, residents/fellows should participate<br><br>17. Some form of endometriosis specific quality of life score/questionnaire should be collected/documented.<br><br>18. Data should be collected at the MDT, preferably by national or international registry (if available)<br><br>19. Follow-up should be defined.<br><br>20. For most clinics, there should be one general MDT. Different MDTs (e.g. fertility, imaging, pain etc.) exceed resources.<br><br>21. External physicians should present their cases.	Psychologist, Sexologist, Thoracic Surgeon, Neurosurgeon.<br><br>20. MDTs should be multiprofessional (including other professions than doctors).<br><br>21. Following other professions should be usually present: Endometriosis nurse, Physiotherapist.<br><br>22. (Following other professions should be present on demand: Occupational Therapist, Nutritionist, Social worker.)<br><br>23. More complex cases should be discussed at MDTs before and after therapy, but this can be decided on an individual basis (not every case needs pre- and post-therapy discussion).<br><br>24. MDTs should be used for teaching, residents/fellows should participate whenever possible.<br><br>25. Some form of endometriosis specific

22. Presenter: treating physician (alternatively residents or fellows)

23. Constant Team reviewing cases before presentation.

24. Possibility of patients to participate in the MDT.

score/questionnaire (e.g. WERF questionnaire) should be collected/documented.

26. Data should be collected from cases discussed at the MDT.

27. Data should be collected preferably by means of a national or international registry, alternatively, by a registry for MDT cases or then the clinic’s information system.

28. Follow-up should be defined at the MDT.

29. For most clinics, there should be one general MDT. Holding different MDTs (e.g. fertility, imaging, pain etc.) exceeds resources in most cases.

30. External physicians should be able to present their cases.

31. The treating physician should present the case, alternatively residents or fellows.

			32. A constant team (e.g. consisting of a senior physician/fellow and an endometriosis nurse) should briefly review the cases before presentation.<br><br>33. (Patients should be able to participate in the MDT during their case discussion.)
Institutions	19. Determine which hospitals should have an MDT (size, centre, academic, etc.)<br><br>20. It should be mandatory for certified endometriosis centres to have an MDT	25. Every university hospital treating endometriosis should have an MDT<br><br>26. Every tertiary referral centre (central hospital, not academic) treating endometriosis should have an MDT<br><br>27. Certified endometriosis centre should have an MDT<br><br>28. All hospitals that operate on endometriosis patients should have access to an MDT<br><br>29. Having an MDT should not depend only on caseload	34. All clinics that provide surgery for endometriosis should have access to an MDT.<br><br>35. Every tertiary referral centre (central hospital, not academic) treating endometriosis patients should have/be part of an MDT.<br><br>36. Every university hospital treating endometriosis patients should have/be part of an MDT.<br><br>37. Endometriosis centres (regardless of whether and how certified) should have/be part of an MDT.

(reference >100 / year, MDT recommended)

30. Treatment of peritoneal endometriosis: access to MDT

31. Treatment of ovarian endometriosis: access to MDT

32. Treatment of deep endometriosis: have own/part of MDT

33. Treatment of more rare manifestations: have own/part of MDT

34. Treatment of adolescent patients: access to MDT

35. Chronic pain treatment (complex pain therapy): access to MDT

36. Treatment of fertility patients: access to MDT

37. Therapy after conservative treatment failure: access to MDT

38. Treatment after surgical treatment failure (redo surgery): have/be part of MDT

38. Having/being a part of an MDT should be a prerequisite for certification of endometriosis centres.

39. Recommendation to have or be part of an MDT should not only depend on caseload (smaller caseloads are likely to be even more dependent on MDTs for selected cases). As a reference, above 100 general cases/year one should have/be part of an MDT.

40. All clinics that treat the following subtype should have access to an MDT: peritoneal endometriosis.

41. All clinics that treat the following subtype should have access to an MDT: ovarian endometriosis.

42. All clinics that treat the following subtype should have/be part of an MDT: deep endometriosis.

43. All clinics that treat the following subtype should have/be part of an MDT: more rare

	39. Complex endometriosis surgical procedures (e.g. bowel resections): have/be part of MDT	manifestations, e.g. thoracic, diaphragmatic endometriosis.<br><br>44. (All clinics that treat adenomyosis should have access to an MDT.)<br><br>45. All clinics that treat adolescent patients should have access to an MDT.<br><br>46. All clinics that provide the following treatment should have access to an MDT: chronic pain treatment (complex/multimodal pain therapy).<br><br>47. All clinics that provide the following treatment should have access to an MDT: fertility therapy.<br><br>48. All clinics that provide the following treatment should have access to an MDT: therapy after conservative treatment failure.<br><br>49. All clinics that provide the following treatment should have/be part of an MDT: therapy

			after surgical treatment failure (redo surgery).<br><br>50. All clinics that provide the following treatment should have/be part of an MDT: more complex endometriosis surgical procedures (e.g. bowel resections).
Patient Selection	21. Selection of cases	40. Only selected endometriosis patients should be discussed at MDT<br><br>41. Selected patients discussed at MDT: peritoneal endometriosis<br><br>42. Selected patients discussed at MDT: ovarian endometriosis<br><br>43. All patients discussed at MDT: deep endometriosis<br><br>44. All patients discussed at MDT: rare manifestations<br><br>45. Selected patients discussed at MDT: chronic pain<br><br>46. Selected adolescent	51. Not every endometriosis patient needs to be discussed at an MDT.<br><br>52. Selected patients, but not necessarily every case, with the following disease manifestation should be discussed at an MDT: peritoneal endometriosis.<br><br>53. Selected patients, but not necessarily every case, with the following disease manifestation should be discussed at an MDT: ovarian endometriosis.<br><br>54. Selected adolescent patients, but not necessarily every case, should be

patients discussed at MDT

47. All patients discussed at MDT: infertility

48. Fertility Therapy at MDT: selected patients

49. Complex Pain Therapy at MDT: all patients

50. Standard endocrine Therapy at MDT: selected patients

51. After failure of second-line therapy or higher: MDT

52. Surgery peritoneal endometriosis at MDT: selected patients

53. Surgery ovarian endometriosis at MDT: selected patients

54. Surgery deep endometriosis at MDT: all patients

55. Complex surgery deep endometriosis (e.g. intestinal manifestations) at MDT: all patients

56. Treatment failure in general at MDT: all patients

discussed at an MDT.

55. Selected patients, but not necessarily every case, with the following disease manifestation should be discussed at an MDT: adenomyosis.

56. All patients with the following disease manifestation should be discussed at an MDT: deep endometriosis.

57. All patients with the following disease manifestation should be discussed at an MDT: more rare manifestations (e.g. diaphragmatic/thoracic endometriosis)

58. Selected patients, but not necessarily every case, with the following disease manifestation should be discussed at an MDT: chronic pain.

59. All patients with the following disease manifestation

57. Diagnostic uncertainties at MDT: all patients

58. Disease recurrence at MDT: all or selected patients?

59. Patients with previous endometriosis surgery at MDT: selected patients

should be discussed at an MDT: infertility.

60. (Selected patients, but not necessarily every case, receiving following therapy should be discussed at an MDT: standard endocrine therapy.)

61. Selected patients, but not necessarily every case, receiving following therapy should be discussed at an MDT: Fertility therapy.

62. All patients receiving following therapy should be discussed at an MDT: complex (multimodal) pain therapy.

63. Endocrine therapy after failure of first-line therapy is not needed to be discussed at an MDT in general. It should though be discussed after failure of second-line therapy or higher.

64. Selected patients, but not necessarily every case, receiving

following therapy should be discussed at an MDT: surgery for peritoneal lesions.

65. Selected patients, but not necessarily every case, receiving following therapy should be discussed at an MDT: endometrioma surgery.

66. All patients receiving following therapy should be discussed at an MDT: deep endometriosis surgery.

67. All patients receiving following therapy should be discussed at an MDT: complex deep endometriosis surgery (e.g. excision of intestinal manifestations)

68. All patients receiving following therapy should be discussed at an MDT: therapy after treatment failure in general.

69. All patients with diagnostic uncertainties

			should be discussed at an MDT.<br><br>70. (All patients with recurrence should be discussed at an MDT.)<br><br>71. Selected patients, but not necessarily every case, with previous endometriosis surgery should be discussed at an MDT.
Imaging Modalities	22. What imaging should be shown<br><br>23. Reviewing Imaging together<br><br>24. Ultrasound<br><br>25. MRI, should be shown by radiologist<br><br>26. Intraoperative Imaging	60. Imaging re-viewed together at MDT<br><br>61. Ultrasound re-viewed together in selected cases<br><br>62. The person who did the ultrasound should show images<br><br>63. MRI re-viewed in all cases<br><br>64. Radiologist should show MRI<br><br>65. Intraoperative imaging re-viewed in selected cases	72. Generally, imaging should be re-viewed together at the MDT.<br><br>73. (Ultrasound imaging should be re-viewed together at the MDT.)<br><br>74. The sonographer should show the ultrasound images.<br><br>75. MR imaging should be re-viewed together at the MDT.<br><br>76. The radiologist should demonstrate the MR imaging.<br><br>77. In selected cases, intraoperative imaging should be re-viewed

			together at the MDT.
Classification	27. Which classifications should be provided<br><br>28. Classification given pre- and post-therapy	66. Recommended classification at MDT: #Enzian. Other classifications can be used additionally.<br><br>67. Classification should be reviewed/discussed at the MDT in selected cases<br><br>68. Classification should be provided pre- and post-therapy at MDT	78. A classification should be routinely used at the MDT.<br><br>79. The classification should be provided pre-therapy on clinical findings as well as on imaging, and then after surgical intervention, if applicable.<br><br>80. The classification should be mentioned both pre- and post-therapy.<br><br>81. The classification should be mentioned at the MDT, reviewed/discussed only in selected cases.<br><br>82. In this consensus, the most recommended classification was #Enzian. Other classifications and scores (rASRM, EFI, The AAGL 2021 Endometriosis Classification etc.) can be used additionally depending on indication and practice.
